# Ethane-1,2-diaminium bis­{5-[4-(1*H*-tetra­zol-5-yl)phen­yl]tetra­zolide} dihydrate

**DOI:** 10.1107/S1600536811034143

**Published:** 2011-08-27

**Authors:** Chun-Rong Li, Zheng-Qiang Xia

**Affiliations:** a School of Environmental Science and Engineering, Chang’an University, Xi’an 710054, Shaanxi, People’s Republic of China; b College of Chemistry and Materials Science, Northwest University, Xi’an 710069, Shaanxi, People’s Republic of China

## Abstract

In the two anions of the title salt, C_2_H_10_N_2_^2+^·2C_8_H_5_N_8_^−^·2H_2_O, the central aromatic rings make dihedral angles of 13.53 (6) and 6.53 (7)° with the deprotonated tetra­zole rings, and 11.39 (6) and 10.41 (9)° with the other tetra­zole groups. In the crystal, the cations, anions and water mol­ecules are linked by an extensive O—H⋯N, N—H⋯O and N—H⋯N hydrogen-bond network into two-dimensional wave-like duplex sheets extending parallel to the *bc* plane. π–π stacking inter­actions between benzene rings [inter­centroid distances are 3.8482 (4) and 3.9621 (5) Å] and between tetra­zole rings [inter­centroid distances are 3.4350 (4) and 3.7169 (4) Å] further consolidate the crystal structure.

## Related literature

For similar structures, see: Tao *et al.* (2004 ▶); Deng *et al.* (2010 ▶); He *et al.* (2008 ▶). For 5,5′-(1,4-phenyl­ene)bis­(1*H*-tetra­zole) applied in coordination chemistry, see: Liu *et al.* (2010 ▶); Ouellette *et al.* (2009 ▶); Dinca *et al.* (2006 ▶); Qiao *et al.* (2011 ▶).
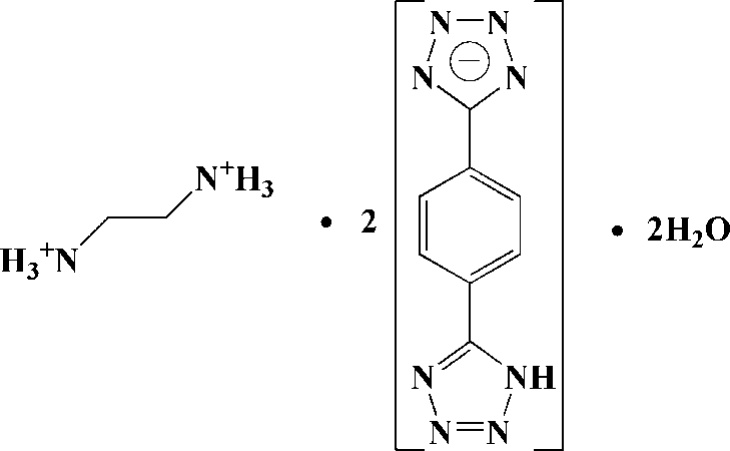


## Experimental

### 

#### Crystal data

C_2_H_10_N_2_^2+^·2C_8_H_5_N_8_^−^·2H_2_O
*M* *_r_* = 524.55
Triclinic, 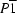

*a* = 7.3918 (9) Å
*b* = 12.4699 (16) Å
*c* = 13.6367 (17) Å
α = 89.774 (2)°
β = 78.556 (2)°
γ = 74.153 (2)°
*V* = 1183.5 (3) Å^3^
*Z* = 2
Mo *K*α radiation
μ = 0.11 mm^−1^
*T* = 296 K
0.32 × 0.28 × 0.11 mm

#### Data collection

Bruker APEXII CCD diffractometer
Absorption correction: multi-scan (*SADABS*; Bruker, 2008 ▶) *T*_min_ = 0.966, *T*_max_ = 0.988
5924 measured reflections
4089 independent reflections
3254 reflections with *I* > 2σ(*I*)
*R*_int_ = 0.017

#### Refinement

*R*[*F* ^2^ > 2σ(*F* ^2^)] = 0.042
*wR*(*F* ^2^) = 0.122
*S* = 1.02
4089 reflections
345 parameters
H-atom parameters constrained
Δρ_max_ = 0.19 e Å^−3^
Δρ_min_ = −0.23 e Å^−3^

### 

Data collection: *APEX2* (Bruker, 2008 ▶); cell refinement: *SAINT* (Bruker, 2008 ▶); data reduction: *SAINT*; program(s) used to solve structure: *SHELXS97* (Sheldrick, 2008 ▶); program(s) used to refine structure: *SHELXL97* (Sheldrick, 2008 ▶); molecular graphics: *ORTEP-3* (Farrugia, 1997 ▶); software used to prepare material for publication: *SHELXL97*.

## Supplementary Material

Crystal structure: contains datablock(s) global, I. DOI: 10.1107/S1600536811034143/yk2016sup1.cif

Structure factors: contains datablock(s) I. DOI: 10.1107/S1600536811034143/yk2016Isup2.hkl

Supplementary material file. DOI: 10.1107/S1600536811034143/yk2016Isup3.cml

Additional supplementary materials:  crystallographic information; 3D view; checkCIF report

## Figures and Tables

**Table 1:** Hydrogen-bond geometry (Å, °)

*D*—H⋯*A*	*D*—H	H⋯*A*	*D*⋯*A*	*D*—H⋯*A*
O2—H2*B*⋯N4*A*^i^	0.82	2.02	2.843 (2)	177
O2—H2*A*⋯N13*A*	0.85	2.08	2.919 (2)	173
O1—H1*A*⋯N4*B*	0.84	2.02	2.857 (2)	179
O1—H1*B*⋯N13*B*^ii^	0.85	2.10	2.946 (2)	174
N10—H10*E*⋯N3*A*^iii^	0.89	2.02	2.869 (2)	160
N10—H10*D*⋯N1*B*^iv^	0.89	2.00	2.848 (2)	159
N10—H10*C*⋯N14*A*^v^	0.89	2.08	2.938 (2)	163
N9—H9*E*⋯N1*A*^vi^	0.89	1.98	2.8517 (19)	165
N9—H9*D*⋯N14*B*^ii^	0.89	2.13	2.888 (2)	143
N9—H9*C*⋯N3*B*	0.89	2.01	2.856 (2)	159
N11*B*—H11*B*⋯O2	0.86	1.86	2.685 (2)	161
N11*A*—H11*A*⋯O1	0.86	1.87	2.6903 (19)	160

Symmetry codes: (i) 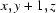; (ii) 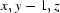; (iii) 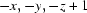; (iv) 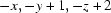; (v) 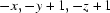; (vi) 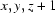.

**Fig. 1.**
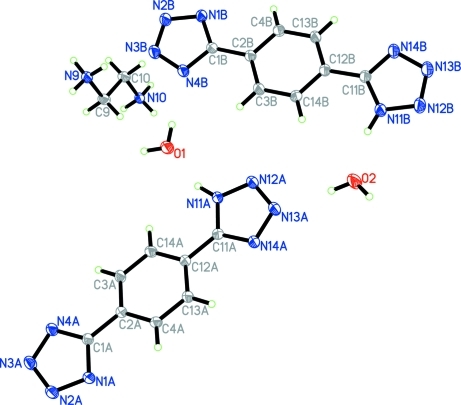
Molecular structure of the title compound, showing the atom labeling scheme and displacement ellipsoids drawn at the 30% probalility level.

**Fig. 2.**
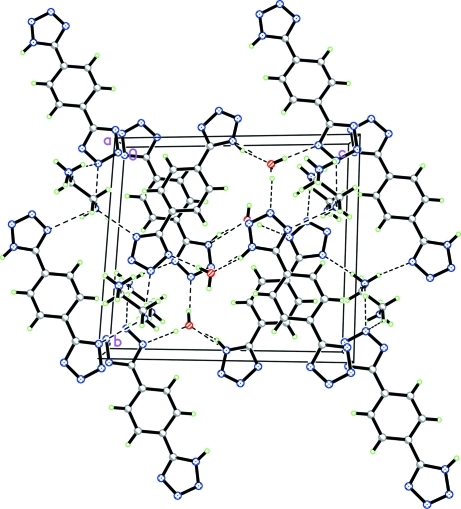
A view of the crystal packing of the title compound, showing the O—H···N, N—H···O and N—H···N hydrogen bonds interactions. Symmetry operators: ix, y + 1, z; iix, y - 1, z; iii -x, -y, -z + 1; iv -x, -y + 1, -z + 2; v -x, -y + 1, -z + 1; vix, y, z + 1.

## supplementary crystallographic information

### Comment

Recently, 5,5'-(1,4-phenylene)-bis(1*H*-tetrazole) has been widely employed in the construction of many useful metal-organic frameworks (Liu *et al.*, 2010; Ouellette *et al.*, 2009; Dinca *et al.*, 2006). This compound attracted our attention and our recent investigation on it (Qiao *et al.*, 2011) has revealed its potential applications in energetic materials as the additives for the propellant. However, reports on its use in the construction of co-crystals are very scarce. Here, in the reaction of ethylenediamine, 5,5'-(1,4-phenylene)bis(1*H*-tetrazole) and PbCl_2_ under hydrothermal conditions, we have unexpectedly obtained the title compound, C_2_H_10_N_2_^2+^.2C_8_H_5_N_8_^-^.2H_2_O, and determined its crystal structure.

The asymmetric unit of the title salt is composed of one ethylenediaminium cation, two 5-[4-(1*H*-tetrazol-5-yl)phenyl]tetrazolide monoanions and two water molecules (Fig.1). Both the amine N atoms of the ethylenediaminium cation are protonated. The geometric parameters are within the normal ranges.

In the crystal structure, the two terminal tetrazole rings of the anions are nearly coplanar with the dihedral angles of 5.03 (7) or 6.37 (10)°. It is noteworthy that there are two types of π-π stacking interactions: one occurs between benzene rings with centroid-centroid distances of 3.8482 (4) and 3.9621 (5) Å, the other occurs between tetrazole rings with centroid-centroid distances of 3.4350 (4) and 3.7169 (4) Å. Thus, a wide range of hydrogen bonds (O—H···N, N—H···O and N—H···N) (Table 1) and π-π stacking interactions contribute to the formation of the supramolecular network (Fig. 2).

### Experimental

Lead chloride (0.0278 g, 0.1 mmol) and 5,5'-(1,4-phenylene)-bis(1*H*-tetrazole) (0.0215 g, 0.1 mmol) were added to water (6 ml). The pH of this solution was adjusted to neutral with ethylenediamine solution. The solution was sealed in a 10-ml Teflon-lined stainless reactor at 393 K for 3 days. After the sample was cooled to room temperature at a rate of 5 K/h, the colorless block crystals suitable for X-ray analysis were obtained.

### Refinement

All H atoms attached to C atoms were fixed geometrically and treated as riding with C—H = 0.97 (methylene) and 0.93 Å (aromatic), *U*_iso_(H) = 1.2Ueq(C). The H atoms bonded to N atoms were placed in calculated positions and refined in riding mode with N—H = 0.86 (tetrazole) and 0.89 Å (amine), *U*_iso_(H) = 1.2Ueq(N of tetrazole), *U*_iso_(H) = 1.5Ueq(N of amine). The water H atoms were located in difference Fourier maps, with distance restraints of O—H = 0.84±0.02 Å, and then refined with isotropic thermal parameters 1.5 times those of O atoms.

### Crystal data

**Table:**

C_2_H_10_N_2_^2+^·2C_8_H_5_N_8_^−^·2H_2_O	*Z* = 2
*M**_r_* = 524.55	*F*(000) = 548
Triclinic, *P*1	*D*_x_ = 1.472 Mg m^−^^3^*D*_m_ = 1.472 Mg m^−^^3^*D*_m_ measured by not measured
Hall symbol: -P 1	Mo *K*α radiation, λ = 0.71073 Å
*a* = 7.3918 (9) Å	Cell parameters from 2314 reflections
*b* = 12.4699 (16) Å	θ = 2.9–25.8°
*c* = 13.6367 (17) Å	µ = 0.11 mm^−^^1^
α = 89.774 (2)°	*T* = 296 K
β = 78.556 (2)°	Block, colorless
γ = 74.153 (2)°	0.32 × 0.28 × 0.11 mm
*V* = 1183.5 (3) Å^3^	

### Data collection

**Table:**

Bruker APEXII CCD diffractometer	4089 independent reflections
Radiation source: fine-focus sealed tube	3254 reflections with *I* > 2σ(*I*)
graphite	*R*_int_ = 0.017
φ and ω scans	θ_max_ = 25.0°, θ_min_ = 1.5°
Absorption correction: multi-scan (*SADABS*; Bruker, 2008)	*h* = −8→8
*T*_min_ = 0.966, *T*_max_ = 0.988	*k* = −14→11
5924 measured reflections	*l* = −16→16

### Refinement

**Table:**

Refinement on *F*^2^	Primary atom site location: structure-invariant direct methods
Least-squares matrix: full	Secondary atom site location: difference Fourier map
*R*[*F*^2^ > 2σ(*F*^2^)] = 0.042	Hydrogen site location: inferred from neighbouring sites
*wR*(*F*^2^) = 0.122	H-atom parameters constrained
*S* = 1.02	*w* = 1/[σ^2^(*F*_o_^2^) + (0.0641*P*)^2^ + 0.2582*P*] where *P* = (*F*_o_^2^ + 2*F*_c_^2^)/3
4089 reflections	(Δ/σ)_max_ < 0.001
345 parameters	Δρ_max_ = 0.19 e Å^−^^3^
0 restraints	Δρ_min_ = −0.23 e Å^−^^3^

### Special details

**Table:**

Geometry. All s.u.'s (except the s.u. in the dihedral angle between two l.s. planes) are estimated using the full covariance matrix. The cell s.u.'s are taken into account individually in the estimation of s.u.'s in distances, angles and torsion angles; correlations between s.u.'s in cell parameters are only used when they are defined by crystal symmetry. An approximate (isotropic) treatment of cell s.u.'s is used for estimating s.u.'s involving l.s. planes.
Refinement. Refinement of *F*^2^ against ALL reflections. The weighted *R*-factor *wR* and goodness of fit *S* are based on *F*^2^, conventional *R*-factors *R* are based on *F*, with *F* set to zero for negative *F*^2^. The threshold expression of *F*^2^ > σ(*F*^2^) is used only for calculating *R*-factors(gt) *etc*. and is not relevant to the choice of reflections for refinement. *R*-factors based on *F*^2^ are statistically about twice as large as those based on *F*, and *R*- factors based on ALL data will be even larger.

### Fractional atomic coordinates and isotropic or equivalent isotropic displacement parameters (Å^2^)

**Table:**

	*x*	*y*	*z*	*U*_iso_*/*U*_eq_	
C11A	0.2135 (2)	0.47786 (13)	0.31175 (12)	0.0301 (4)	
C12A	0.2209 (2)	0.37261 (13)	0.26227 (12)	0.0297 (4)	
C13A	0.2551 (3)	0.36244 (14)	0.15792 (12)	0.0333 (4)	
H13A	0.2671	0.4237	0.1207	0.040*	
C14A	0.2017 (3)	0.28026 (14)	0.31627 (13)	0.0367 (4)	
H14A	0.1790	0.2857	0.3859	0.044*	
C11B	0.2752 (3)	0.96745 (14)	0.60365 (12)	0.0333 (4)	
C12B	0.2807 (3)	0.86208 (14)	0.65171 (12)	0.0321 (4)	
C13B	0.2756 (3)	0.85759 (14)	0.75418 (13)	0.0352 (4)	
H13B	0.2664	0.9219	0.7914	0.042*	
C14B	0.2936 (3)	0.76531 (15)	0.59755 (13)	0.0394 (5)	
H14B	0.2969	0.7672	0.5290	0.047*	
C9	0.0711 (3)	0.20961 (15)	0.83423 (13)	0.0378 (4)	
H9A	0.0183	0.1467	0.8323	0.045*	
H9B	0.0523	0.2512	0.7751	0.045*	
C10	−0.0329 (3)	0.28374 (15)	0.92698 (13)	0.0370 (4)	
H10A	−0.0148	0.2421	0.9862	0.044*	
H10B	0.0201	0.3466	0.9290	0.044*	
C1A	0.2689 (2)	0.06357 (13)	0.11169 (12)	0.0285 (4)	
C2A	0.2516 (2)	0.16970 (13)	0.16380 (12)	0.0289 (4)	
C3A	0.2159 (3)	0.18057 (14)	0.26764 (13)	0.0357 (4)	
H3A	0.2012	0.1198	0.3050	0.043*	
C4A	0.2713 (3)	0.26267 (14)	0.10956 (12)	0.0325 (4)	
H4A	0.2956	0.2570	0.0399	0.039*	
C1B	0.3137 (2)	0.55566 (14)	0.79614 (12)	0.0294 (4)	
C2B	0.2975 (2)	0.66181 (14)	0.74691 (12)	0.0308 (4)	
C3B	0.3016 (3)	0.66656 (15)	0.64432 (13)	0.0391 (4)	
H3B	0.3099	0.6024	0.6071	0.047*	
C4B	0.2841 (3)	0.75917 (14)	0.80075 (12)	0.0343 (4)	
H4B	0.2808	0.7575	0.8693	0.041*	
N11A	0.2160 (2)	0.49535 (11)	0.40801 (10)	0.0373 (4)	
H11A	0.2204	0.4457	0.4521	0.045*	
N12A	0.2104 (3)	0.60166 (12)	0.42518 (11)	0.0446 (4)	
N13A	0.2043 (3)	0.64840 (12)	0.34094 (11)	0.0426 (4)	
N14A	0.2065 (2)	0.57367 (12)	0.26830 (10)	0.0355 (4)	
N11B	0.2501 (3)	0.98954 (12)	0.51052 (11)	0.0436 (4)	
H11B	0.2368	0.9431	0.4679	0.052*	
N12B	0.2491 (3)	1.09568 (13)	0.49410 (12)	0.0527 (5)	
N13B	0.2731 (3)	1.13653 (13)	0.57554 (12)	0.0506 (5)	
N14B	0.2906 (3)	1.05895 (12)	0.64532 (11)	0.0417 (4)	
N9	0.2778 (2)	0.16876 (11)	0.83363 (10)	0.0322 (3)	
H9C	0.3286	0.2261	0.8290	0.048*	
H9D	0.3353	0.1206	0.7815	0.048*	
H9E	0.2945	0.1350	0.8901	0.048*	
N10	−0.2399 (2)	0.32474 (11)	0.92681 (10)	0.0315 (3)	
H10C	−0.2562	0.3631	0.8726	0.047*	
H10D	−0.2994	0.3688	0.9814	0.047*	
H10E	−0.2888	0.2670	0.9263	0.047*	
N1A	0.2669 (2)	0.05241 (11)	0.01421 (10)	0.0339 (4)	
N2A	0.2887 (2)	−0.05668 (12)	−0.00421 (11)	0.0381 (4)	
N3A	0.3040 (2)	−0.10827 (12)	0.07894 (11)	0.0392 (4)	
N4A	0.2908 (2)	−0.03469 (12)	0.15402 (10)	0.0351 (4)	
N1B	0.3320 (2)	0.54178 (11)	0.89155 (10)	0.0346 (4)	
N2B	0.3495 (2)	0.43315 (12)	0.90616 (11)	0.0385 (4)	
N3B	0.3432 (2)	0.38426 (12)	0.82212 (11)	0.0404 (4)	
N4B	0.3198 (2)	0.45938 (12)	0.75156 (11)	0.0376 (4)	
O1	0.2556 (2)	0.37535 (11)	0.57068 (10)	0.0567 (4)	
H1B	0.2661	0.3062	0.5750	0.085*	
H1A	0.2766	0.4000	0.6234	0.085*	
O2	0.2347 (3)	0.87668 (13)	0.34653 (11)	0.0842 (7)	
H2A	0.2303	0.8101	0.3399	0.126*	
H2B	0.2537	0.9038	0.2915	0.126*	

### Atomic displacement parameters (Å^2^)

**Table:**

	*U*^11^	*U*^22^	*U*^33^	*U*^12^	*U*^13^	*U*^23^
C11A	0.0383 (10)	0.0262 (9)	0.0257 (8)	−0.0075 (7)	−0.0084 (7)	0.0014 (7)
C12A	0.0370 (10)	0.0251 (9)	0.0269 (9)	−0.0077 (7)	−0.0081 (7)	0.0007 (7)
C13A	0.0496 (11)	0.0261 (9)	0.0284 (9)	−0.0146 (8)	−0.0120 (8)	0.0040 (7)
C14A	0.0569 (12)	0.0319 (10)	0.0227 (8)	−0.0146 (8)	−0.0084 (8)	0.0012 (7)
C11B	0.0463 (11)	0.0281 (9)	0.0252 (9)	−0.0100 (8)	−0.0069 (7)	0.0005 (7)
C12B	0.0423 (11)	0.0261 (9)	0.0287 (9)	−0.0099 (7)	−0.0087 (7)	0.0024 (7)
C13B	0.0513 (12)	0.0265 (9)	0.0292 (9)	−0.0122 (8)	−0.0096 (8)	0.0003 (7)
C14B	0.0643 (13)	0.0319 (10)	0.0253 (9)	−0.0141 (9)	−0.0157 (8)	0.0038 (7)
C9	0.0398 (11)	0.0394 (10)	0.0331 (10)	−0.0069 (8)	−0.0104 (8)	−0.0061 (8)
C10	0.0381 (11)	0.0406 (10)	0.0323 (9)	−0.0104 (8)	−0.0083 (8)	−0.0046 (8)
C1A	0.0347 (10)	0.0261 (9)	0.0266 (8)	−0.0115 (7)	−0.0069 (7)	0.0029 (7)
C2A	0.0351 (10)	0.0244 (8)	0.0292 (9)	−0.0092 (7)	−0.0099 (7)	0.0010 (7)
C3A	0.0563 (12)	0.0252 (9)	0.0284 (9)	−0.0167 (8)	−0.0077 (8)	0.0050 (7)
C4A	0.0459 (11)	0.0307 (9)	0.0240 (8)	−0.0135 (8)	−0.0103 (7)	0.0029 (7)
C1B	0.0352 (10)	0.0272 (9)	0.0279 (9)	−0.0107 (7)	−0.0083 (7)	0.0014 (7)
C2B	0.0368 (10)	0.0292 (9)	0.0279 (9)	−0.0109 (7)	−0.0081 (7)	0.0020 (7)
C3B	0.0630 (13)	0.0266 (9)	0.0304 (9)	−0.0137 (9)	−0.0142 (9)	−0.0006 (7)
C4B	0.0496 (11)	0.0312 (9)	0.0230 (8)	−0.0126 (8)	−0.0074 (8)	0.0014 (7)
N11A	0.0625 (11)	0.0240 (8)	0.0287 (8)	−0.0130 (7)	−0.0160 (7)	0.0025 (6)
N12A	0.0762 (12)	0.0290 (8)	0.0331 (8)	−0.0175 (8)	−0.0179 (8)	0.0004 (7)
N13A	0.0665 (12)	0.0287 (8)	0.0354 (9)	−0.0157 (7)	−0.0134 (8)	−0.0017 (7)
N14A	0.0526 (10)	0.0267 (8)	0.0292 (8)	−0.0123 (7)	−0.0116 (7)	0.0017 (6)
N11B	0.0780 (13)	0.0284 (8)	0.0289 (8)	−0.0186 (8)	−0.0163 (8)	0.0032 (6)
N12B	0.0961 (15)	0.0315 (9)	0.0346 (9)	−0.0219 (9)	−0.0170 (9)	0.0077 (7)
N13B	0.0888 (14)	0.0299 (9)	0.0341 (9)	−0.0189 (9)	−0.0116 (9)	0.0036 (7)
N14B	0.0689 (12)	0.0283 (8)	0.0296 (8)	−0.0165 (7)	−0.0098 (7)	0.0032 (6)
N9	0.0397 (9)	0.0283 (8)	0.0284 (7)	−0.0087 (6)	−0.0074 (6)	−0.0002 (6)
N10	0.0387 (9)	0.0260 (7)	0.0297 (8)	−0.0083 (6)	−0.0078 (6)	0.0008 (6)
N1A	0.0479 (9)	0.0276 (8)	0.0287 (8)	−0.0122 (7)	−0.0111 (7)	0.0003 (6)
N2A	0.0535 (10)	0.0304 (8)	0.0324 (8)	−0.0151 (7)	−0.0086 (7)	−0.0019 (6)
N3A	0.0553 (10)	0.0283 (8)	0.0365 (9)	−0.0177 (7)	−0.0064 (7)	−0.0013 (7)
N4A	0.0505 (10)	0.0274 (8)	0.0303 (8)	−0.0153 (7)	−0.0083 (7)	0.0024 (6)
N1B	0.0485 (10)	0.0287 (8)	0.0281 (8)	−0.0123 (7)	−0.0092 (7)	0.0045 (6)
N2B	0.0539 (10)	0.0309 (8)	0.0347 (8)	−0.0161 (7)	−0.0122 (7)	0.0074 (6)
N3B	0.0562 (10)	0.0301 (8)	0.0379 (9)	−0.0161 (7)	−0.0106 (7)	0.0042 (7)
N4B	0.0576 (10)	0.0281 (8)	0.0320 (8)	−0.0170 (7)	−0.0135 (7)	0.0039 (6)
O1	0.1072 (13)	0.0371 (8)	0.0373 (7)	−0.0283 (8)	−0.0303 (8)	0.0085 (6)
O2	0.190 (2)	0.0513 (10)	0.0361 (8)	−0.0628 (12)	−0.0398 (11)	0.0109 (7)

### Geometric parameters (Å, °)

**Table:**

C11A—N14A	1.324 (2)	C1B—N4B	1.334 (2)
C11A—N11A	1.336 (2)	C1B—N1B	1.340 (2)
C11A—C12A	1.461 (2)	C1B—C2B	1.468 (2)
C12A—C14A	1.389 (2)	C2B—C4B	1.391 (2)
C12A—C13A	1.395 (2)	C2B—C3B	1.395 (2)
C13A—C4A	1.375 (2)	C3B—H3B	0.9300
C13A—H13A	0.9300	C4B—H4B	0.9300
C14A—C3A	1.380 (2)	N11A—N12A	1.335 (2)
C14A—H14A	0.9300	N11A—H11A	0.8600
C11B—N14B	1.319 (2)	N12A—N13A	1.289 (2)
C11B—N11B	1.336 (2)	N13A—N14A	1.3574 (19)
C11B—C12B	1.460 (2)	N11B—N12B	1.340 (2)
C12B—C14B	1.388 (2)	N11B—H11B	0.8600
C12B—C13B	1.392 (2)	N12B—N13B	1.287 (2)
C13B—C4B	1.372 (2)	N13B—N14B	1.353 (2)
C13B—H13B	0.9300	N9—H9C	0.8900
C14B—C3B	1.377 (2)	N9—H9D	0.8900
C14B—H14B	0.9300	N9—H9E	0.8900
C9—N9	1.471 (2)	N10—H10C	0.8900
C9—C10	1.510 (2)	N10—H10D	0.8900
C9—H9A	0.9700	N10—H10E	0.8900
C9—H9B	0.9700	N1A—N2A	1.3440 (19)
C10—N10	1.475 (2)	N2A—N3A	1.311 (2)
C10—H10A	0.9700	N3A—N4A	1.349 (2)
C10—H10B	0.9700	N1B—N2B	1.3430 (19)
C1A—N4A	1.336 (2)	N2B—N3B	1.315 (2)
C1A—N1A	1.340 (2)	N3B—N4B	1.3431 (19)
C1A—C2A	1.467 (2)	O1—H1B	0.8477
C2A—C3A	1.388 (2)	O1—H1A	0.8408
C2A—C4A	1.398 (2)	O2—H2A	0.8464
C3A—H3A	0.9300	O2—H2B	0.8243
C4A—H4A	0.9300		
			
N14A—C11A—N11A	107.27 (14)	C2A—C4A—H4A	119.6
N14A—C11A—C12A	125.89 (14)	N4B—C1B—N1B	110.77 (14)
N11A—C11A—C12A	126.82 (15)	N4B—C1B—C2B	124.99 (14)
C14A—C12A—C13A	118.65 (15)	N1B—C1B—C2B	124.19 (15)
C14A—C12A—C11A	121.72 (15)	C4B—C2B—C3B	118.44 (15)
C13A—C12A—C11A	119.58 (14)	C4B—C2B—C1B	120.84 (15)
C4A—C13A—C12A	120.61 (15)	C3B—C2B—C1B	120.68 (15)
C4A—C13A—H13A	119.7	C14B—C3B—C2B	120.64 (16)
C12A—C13A—H13A	119.7	C14B—C3B—H3B	119.7
C3A—C14A—C12A	120.65 (16)	C2B—C3B—H3B	119.7
C3A—C14A—H14A	119.7	C13B—C4B—C2B	120.90 (15)
C12A—C14A—H14A	119.7	C13B—C4B—H4B	119.5
N14B—C11B—N11B	107.58 (15)	C2B—C4B—H4B	119.5
N14B—C11B—C12B	125.96 (15)	N12A—N11A—C11A	109.55 (14)
N11B—C11B—C12B	126.46 (15)	N12A—N11A—H11A	125.2
C14B—C12B—C13B	118.83 (15)	C11A—N11A—H11A	125.2
C14B—C12B—C11B	121.69 (15)	N13A—N12A—N11A	106.35 (14)
C13B—C12B—C11B	119.47 (15)	N12A—N13A—N14A	110.58 (14)
C4B—C13B—C12B	120.58 (16)	C11A—N14A—N13A	106.25 (13)
C4B—C13B—H13B	119.7	C11B—N11B—N12B	109.11 (14)
C12B—C13B—H13B	119.7	C11B—N11B—H11B	125.4
C3B—C14B—C12B	120.60 (16)	N12B—N11B—H11B	125.4
C3B—C14B—H14B	119.7	N13B—N12B—N11B	106.27 (14)
C12B—C14B—H14B	119.7	N12B—N13B—N14B	110.78 (14)
N9—C9—C10	110.58 (14)	C11B—N14B—N13B	106.26 (14)
N9—C9—H9A	109.5	C9—N9—H9C	109.5
C10—C9—H9A	109.5	C9—N9—H9D	109.5
N9—C9—H9B	109.5	H9C—N9—H9D	109.5
C10—C9—H9B	109.5	C9—N9—H9E	109.5
H9A—C9—H9B	108.1	H9C—N9—H9E	109.5
N10—C10—C9	110.26 (14)	H9D—N9—H9E	109.5
N10—C10—H10A	109.6	C10—N10—H10C	109.5
C9—C10—H10A	109.6	C10—N10—H10D	109.5
N10—C10—H10B	109.6	H10C—N10—H10D	109.5
C9—C10—H10B	109.6	C10—N10—H10E	109.5
H10A—C10—H10B	108.1	H10C—N10—H10E	109.5
N4A—C1A—N1A	110.91 (14)	H10D—N10—H10E	109.5
N4A—C1A—C2A	124.96 (14)	C1A—N1A—N2A	105.64 (13)
N1A—C1A—C2A	124.13 (14)	N3A—N2A—N1A	108.65 (13)
C3A—C2A—C4A	118.34 (15)	N2A—N3A—N4A	110.11 (13)
C3A—C2A—C1A	121.17 (14)	C1A—N4A—N3A	104.69 (13)
C4A—C2A—C1A	120.49 (15)	C1B—N1B—N2B	105.69 (13)
C14A—C3A—C2A	120.94 (16)	N3B—N2B—N1B	108.54 (13)
C14A—C3A—H3A	119.5	N2B—N3B—N4B	109.96 (13)
C2A—C3A—H3A	119.5	C1B—N4B—N3B	105.03 (13)
C13A—C4A—C2A	120.80 (15)	H1B—O1—H1A	108.9
C13A—C4A—H4A	119.6	H2A—O2—H2B	110.7

### Hydrogen-bond geometry (Å, °)

**Table:**

*D*—H···*A*	*D*—H	H···*A*	*D*···*A*	*D*—H···*A*
O2—H2B···N4A^i^	0.82	2.02	2.843 (2)	177.
O2—H2A···N13A	0.85	2.08	2.919 (2)	173.
O1—H1A···N4B	0.84	2.02	2.857 (2)	179.
O1—H1B···N13B^ii^	0.85	2.10	2.946 (2)	174.
N10—H10E···N3A^iii^	0.89	2.02	2.869 (2)	160.
N10—H10D···N1B^iv^	0.89	2.00	2.848 (2)	159.
N10—H10C···N14A^v^	0.89	2.08	2.938 (2)	163.
N9—H9E···N1A^vi^	0.89	1.98	2.8517 (19)	165.
N9—H9D···N14B^ii^	0.89	2.13	2.888 (2)	143.
N9—H9C···N3B	0.89	2.01	2.856 (2)	159.
N11B—H11B···O2	0.86	1.86	2.685 (2)	161.
N11A—H11A···O1	0.86	1.87	2.6903 (19)	160.

Symmetry codes: (i) *x*, *y*+1, *z*; (ii) *x*, *y*−1, *z*; (iii) −*x*, −*y*, −*z*+1; (iv) −*x*, −*y*+1, −*z*+2; (v) −*x*, −*y*+1, −*z*+1; (vi) *x*, *y*, *z*+1.
